# Can the Energy Gap in the Protein-Ligand Binding Energy Landscape Be Used as a Descriptor in Virtual Ligand Screening?

**DOI:** 10.1371/journal.pone.0046532

**Published:** 2012-10-10

**Authors:** Arsen V. Grigoryan, Hong Wang, Timothy J. Cardozo

**Affiliations:** Department of Biochemistry and Molecular Pharmacology, New York University School of Medicine, New York, New York, United States of America; Bioinformatics Institute, Singapore

## Abstract

The ranking of scores of individual chemicals within a large screening library is a crucial step in virtual screening (VS) for drug discovery. Previous studies showed that the quality of protein-ligand recognition can be improved using spectrum properties and the shape of the binding energy landscape. Here, we investigate whether the energy gap, defined as the difference between the lowest energy pose generated by a docking experiment and the average energy of all other generated poses and inferred to be a measure of the binding energy landscape sharpness, can improve the separation power between true binders and decoys with respect to the use of the best docking score. We performed retrospective single- and multiple-receptor conformation VS experiments in a diverse benchmark of 40 domains from 38 therapeutically relevant protein targets. Also, we tested the performance of the energy gap on 36 protein targets from the Directory of Useful Decoys (DUD). The results indicate that the energy gap outperforms the best docking score in its ability to discriminate between true binders and decoys, and true binders tend to have larger energy gaps than decoys. Furthermore, we used the energy gap as a descriptor to measure the height of the native binding phase and obtained a significant increase in the success rate of near native binding pose identification when the ligand binding conformations within the boundaries of the native binding phase were considered. The performance of the energy gap was also evaluated on an independent test case of VS-identified PKR-like ER-localized eIF2α kinase (PERK) inhibitors. We found that the energy gap was superior to the best docking score in its ability to more highly rank active compounds from inactive ones. These results suggest that the energy gap of the protein-ligand binding energy landscape is a valuable descriptor for use in VS.

## Introduction

Understanding the mechanism of ligand-receptor recognition plays an important role in the identification of novel compounds for pharmaceutical development [1]–[3]. Docking applies the physico-chemical theory of biomolecular interactions to simulations and is a crucial method for structure-based rational drug discovery [4]. Protein folding and ligand-protein binding phenomena share several physical chemistry characteristics, including a thermodynamically stable native state, large conformational spaces, and complex energy landscapes [3], [5]. Energy landscape analysis was first implemented to investigate protein folding [6]–[17] and was further developed in ligand-protein binding studies [14], [18]–[25]. These studies show that the shape of the binding energy landscape of highly specific protein-ligand complexes has a steep slope towards native state, while less selective complexes have a more uneven shape of the binding energy landscape with low barriers between conformers of the complex [3], [21], [22], [24]. Consideration of intrinsic specificity ratio (ISR), defined as 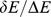, where 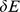 is the energy gap between lowest energy state and average binding energy state (populations of weakly bound states follow Boltzmann distribution), and 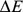 is the energy variance of the weakly binding states, was reported to improve understanding of physiologically relevant ligand recognition by protein receptors [21], [22]. The energy gap has therefore been proposed as a measure of the shape of the energy landscape. The approach suggested in [21], [22] was tested on cyclooxygenase (COX) inhibitors, and known specific inhibitors for COX-2 were correctly identified [22]. It also was shown that consideration of multiple docking solutions significantly increases the success rate of obtaining the crystallographic binding pose [26]–[28]; however the precise range of poses to be considered and the theoretical justification of that range have not yet been proposed. Wei et al [29] used AutoDock in a limited study to investigate the influence of incorporating the ligand-protein binding landscape properties (the energy gap and number of local binding wells) on the success rate of VS against two targets: influenza virus neuraminidase and cyclooxygenase-2. Recently, unguided molecular dynamics simulations of the entire binding process of cancer drug dasatinib and the kinase inhibitor PP1 to Src protein kinase were conducted, and energy landscape analysis was performed [30]. For a review of different aspects related to the nature of ligand-protein binding energy landscapes, as well as various computational approaches to address these aspects see [31].

The goal of this study is to investigate the contribution of intermolecular binding energy landscapes to the quantitative assessment of the quality of ligand recognition by protein receptors on a large and diverse benchmark of therapeutically relevant proteins. Based on studies performed in [21], [22], we evaluated the ability of the energy gap between the lowest energy (best scored pose) and the average energy of all other poses scored in the searched binding energy landscape to discriminate true binders from decoys using single-receptor conformation (SRC) and multiple-receptor conformation (MRC) VS experiments. In this study we used two diverse benchmarks to address this question. The first one consists of 485 X-ray protein conformations of 40 domains from 38 therapeutically relevant protein targets. This benchmark was selected from the flexible Pocketome dataset, which is “an encyclopedia of conformational ensembles of all druggable binding sites that can be identified from co-crystal structures in the PDB” (visit http://pocketome.org/website for details). Henceforth, we will refer to this benchmark as the Pocketome benchmark. The second benchmark consists of 468 X-ray protein conformations of 36 pharmaceutically relevant protein targets from the Directory of Useful Decoys (DUD) [32]–[34]. For comparison purposes, the performance of the best docking score is presented. Also, by using the energy gap as a descriptor we attempt to measure the height of native binding phase and test the success rate of near native binding pose identification when the ligand binding conformations within the boundaries of the native binding phase have been considered. Furthermore, the performance of the energy gap was evaluated on PKR-like ER-localized eIF2α kinase (PERK) inhibitors recently discovered using a homology model as the VS receptor [35]. All simulations were performed with ICM (Molsoft LLC, La Jolla, CA) [36]–[38].

## Materials and Methods

### Protein-ligand Complexes

Two diverse benchmarks were used to investigate the ability of the energy gap to discriminate true binders from decoys. The Pocketome benchmark consists of 485 X-ray conformational ensembles (334 holo structures and 151 apo structures) of 40 domains from 38 therapeutically relevant protein targets (see Table S1 for details) with high resolution and drug-like ligands co-crystallized with these proteins (with 8 ligands per protein domain on average). Four protein domains out of 40 contain only holo structures. The second benchmark used in our study was adopted from [34] and consists of 468 high resolution X-ray protein conformations of 36 pharmaceutically relevant protein targets from the Directory of Useful Decoys (DUD) with 3,611 true binders and 32 decoys per true binder (see Table S8 for details). Because these two benchmarks were originally collected independently, there is an overlap of 7 protein targets between them, however sets of true binders and decoys used in VS experiments against these 7 protein targets were different for each benchmark. The crystal structures were meticulously collected by Irina Kufareva and Ruben Abagyan, UCSD Skaggs School of Pharmacy and Pharmaceutical Sciences [39], [40]. A conformational ensemble for a protein had to represent at least two different crystal structures and include at least one co-crystallized ligand. The atomic coordinates were retrieved from the RCSB Protein Data Bank (PDB) [41].

### Homology Model of PERK

To test the performance of the energy gap in VS against protein homology models we used PERK homology models recently built in our laboratory. Initially, two homology models of the PERK catalytic domain were generated from two crystal structures of eIF2α kinase GCN2 (PDB code: 1zy4 & 1zy5) [42], followed by conformational sampling of the activation loop. This ensemble of multiple receptor structures was used for the subsequent VS (see [35] for details).

### Preparation of Proteins

The receptors were set-up by deleting the chains, heteroatoms, and prosthetic groups not involved in the binding site definition. The protein atom types were assigned, and hydrogen atoms and missing heavy atoms were added. The added or zero occupancy side chains and polar hydrogen atoms were optimized and assigned the lowest energy. Tautomeric states of histidines and the rotations of asparagine and glutamine side chain amidic groups were optimized to improve the hydrogen-bonding patterns. The cognate ligands were deleted from the complexes only after hydrogen optimization.

### Preparation of Ligands

Coordinates of the ligands were extracted either from the crystallographic complexes or according to DUD. Bond orders, tautomeric forms, stereochemistry, hydrogen atoms, and protonation states were assigned automatically by the ICM chemical conversion procedure. Each ligand was assigned the MMFF [43] force field atom types and charges. Ligand molecules were prepared for docking by a rotational search followed by the Cartesian minimization in the absence of the receptor, and the lowest energy conformations were used as starting points for ICM docking.

### Ligand Docking

The well-established ICM docking program was used for the docking calculations. ICM addresses the docking issue as a global optimization problem, implementing a biased probability Monte Carlo (BPMC) global stochastic optimization of the flexible full-atom models of the ligand in the set of grid potential maps representing the protein [37], [38]. These grid energy maps account for the hydrophobic, heavy atom and hydrogen van der Waals interactions, hydrogen-bonding interactions, and electrostatic potential. A diverse set of ligand conformers was first generated from PDB ligand coordinates by ligand sampling *in vacuo*. Each conformer was locally minimized with relaxed bond lengths and bond angles using the MMFF-94 force field in order to remove any bias towards receptor-bound covalent geometry. The generated conformers were then placed into the binding pocket in four principal orientations and used as starting points for Monte Carlo (MC) optimization. The BPMC docking runs were performed using thoroughness setting of 2, which controls the basic number of BPMC steps to be carried out.

### Virtual Screening (VS)

For each docking simulation a stack of diverse binding poses was generated, and their respective scoring energies were evaluated using the ICM scoring function [44]. The score was calculated using the following formula:
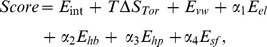
(1)where *E*_vw_, *E*_el_, E_hb_, *E*_hp_, and *E*_sf_ are van der Waals, electrostatic, hydrogen bonding, and nonpolar and polar atom solvation energy differences between bound and unbound states, respectively. *E*_int_ represents the ligand internal strain and Δ*S*_Tor_ is its conformational entropy loss upon binding. The α coefficients are empirical weights that balance the different terms.

For each ligand-receptor pair, three docking runs were performed and 200 binding poses were obtained after each run. All binding poses accumulated after each run were merged in a single conformational stack, and only the geometrically unique solutions, with corresponding energies sorted from low to high, were retained. The energy gap between the lowest energy (best scored pose obtained from three docking runs) and the average energy of all other poses scored in the searched binding energy landscape was calculated and used as a score to discriminate true binders from decoys. If the lowest energy or the average energy of all other poses obtained from the docking runs were found to have a positive value, then those protein-ligand complexes were not included in the final dataset because of possible indication of protein-ligand clashes or other structural anomalies, such as high conformational strain. Performances of the energy gap and the best docking score were evaluated through retrospective SRC and MRC VS experiments aimed at separating known ligand binders from decoys. For the Pocketome benchmark, the evaluation of docking scores and energy gaps for true binders was performed by docking ligands co-crystallized within a given domain to the ligand binding pocket of all conformations of this domain. Henceforth, we will refer to those ligands as true binders. The cognate receptor structures were not included in ensemble docking calculations. The cross-docking effort was aimed at generating diverse decoy complex structures. For every domain, (i) the size range of all cognate (co-crystallized) ligands was determined as the number of their heavy atoms and (ii) 20 ligands chosen randomly from a dataset of ligands of comparable size (±20% of cognate ligand size) were docked into the ligand binding pocket of all conformations of this domain. For DUD benchmark, active ligands (we will refer to those ligands as true binders too) and decoys of a given protein target were docked to the ligand binding pocket of all conformations of this target. The performance of the energy gap was evaluated through MRC VS. For the PERK homology model we tested the performance of the energy gap on 32 compounds, including 20 active and 12 inactive, validated by in vitro kinase inhibition assay [35].

Using the approaches proposed above, the binding energy landscapes of true binders and high-scoring decoys were obtained. We hypothesized that a significant difference between these two landscapes should be observed. The energy gap of the binding energy landscape was used as a score and was evaluated for its ability to distinguish true binders from decoys. The ROC (receiver operating characteristic) curve, or ROC AUC, was used to evaluate the performance of the energy gap. ROC curve analysis [45] describes the ability of a screening method to avoid false positives and false negatives. The ideal screening device demonstrates the ROC AUC value of 1, while a random selection performance corresponds to the ROC AUC value of 0.5. For MRC ensemble runs, the ROC curves were calculated using the best docking score and corresponding energy gap for each ligand obtained from the protein conformations in the ensemble. As an early recognition metric, we used the fraction of true binders recovered within *N* top-ranked hits (both among true binders and decoys) predicted by VS, where *N* is the number of true binders for a given protein target. The discrimination abilities of the energy gap and the best docking score were systematically compared.

### Statistical Analysis

To evaluate the statistical significance of the obtained ROC AUC values, we assumed that they are distributed according to Gaussian distribution with mean 0.5. The uncertainty *σ* is calculated by performing 20 random statistical experiments in which the ranks are reshuffled, calculating individual ROC AUCs and calculating the root mean square deviation of those ROC AUCs. For each ROC AUC value obtained on a given subset of binders and decoys for a given structure, its P-value was calculated as the probability of obtaining the same AUC by random coincidence:
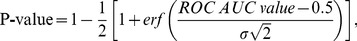
(2)where *erf* is the error function [46] and *σ* is the standard deviation. P-values below 0.05 indicate that the ROC AUC values are statistically significance, while P-values greater than 0.05 indicate that the ROC AUC values are statistically less significant.

### Software and Hardware

The receptor and ligand preparations, the docking simulations, and the energy and gap evaluations were carried out with ICM 3.6-1e (Molsoft LLC, La Jolla, CA). The hardware facility employed in the present study was a 16 64-bit Intel XEON X5560 CPUs Linux-based cluster at New York University School of Medicine (New York, NY). It takes 20–40 seconds to dock one compound into one receptor and perform post docking conformational stack evaluation on a single CPU.

## Results and Discussion

**Figure 1:**
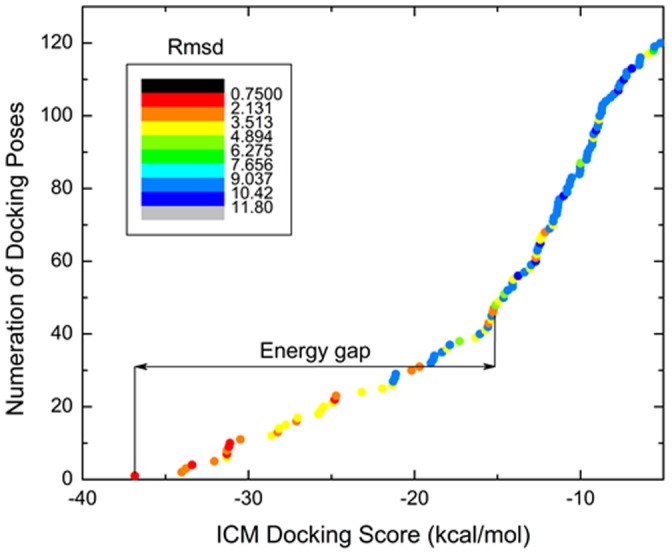
Binding energy landscape evaluation for docking simulation of Chk1 protein kinase inhibitor (PDB HET ID: 422) to Chk1protein kinase (PDB ID: 2br1). Color-coded representation of ICM Docking Scores of all generated docking poses and evaluation of the energy gap. Each dot represents one docking pose of Chk1 protein kinase inhibitor. The dots are colored in different colors spread evenly among rmsd-values from 1.1 to 11.8 Å to the crystallographic position of Chk1 protein kinase inhibitor (PDB HET ID: 422). The lowest ICM Docking Score  = −36.8 kcal/mol (1.1 Å (rmsd) to the crystallographic position), the highest ICM Docking Score  = −5.2 kcal/mol (10 Å (rmsd) to the crystallographic position), and the energy gap of −21.5 kcal/mol.

In this study the data set was utilized to investigate the ability of the energy gap in the searched binding energy landscape to discriminate true binders from decoys. The evaluation of the best docking scores and the energy gaps for true binders and decoys was carried out as is described in the Materials and Methods section. As an example of a binding energy landscape and evaluation of the energy gap, the docking of Chk1 protein kinase inhibitor (PDB HET ID: 422) to Chk1protein kinase (PDB ID: 2br1) is presented in Figure 1.

### Screening Performance of the Energy Gap: Single-Receptor Conformation Virtual Screening

**Figure 2:**
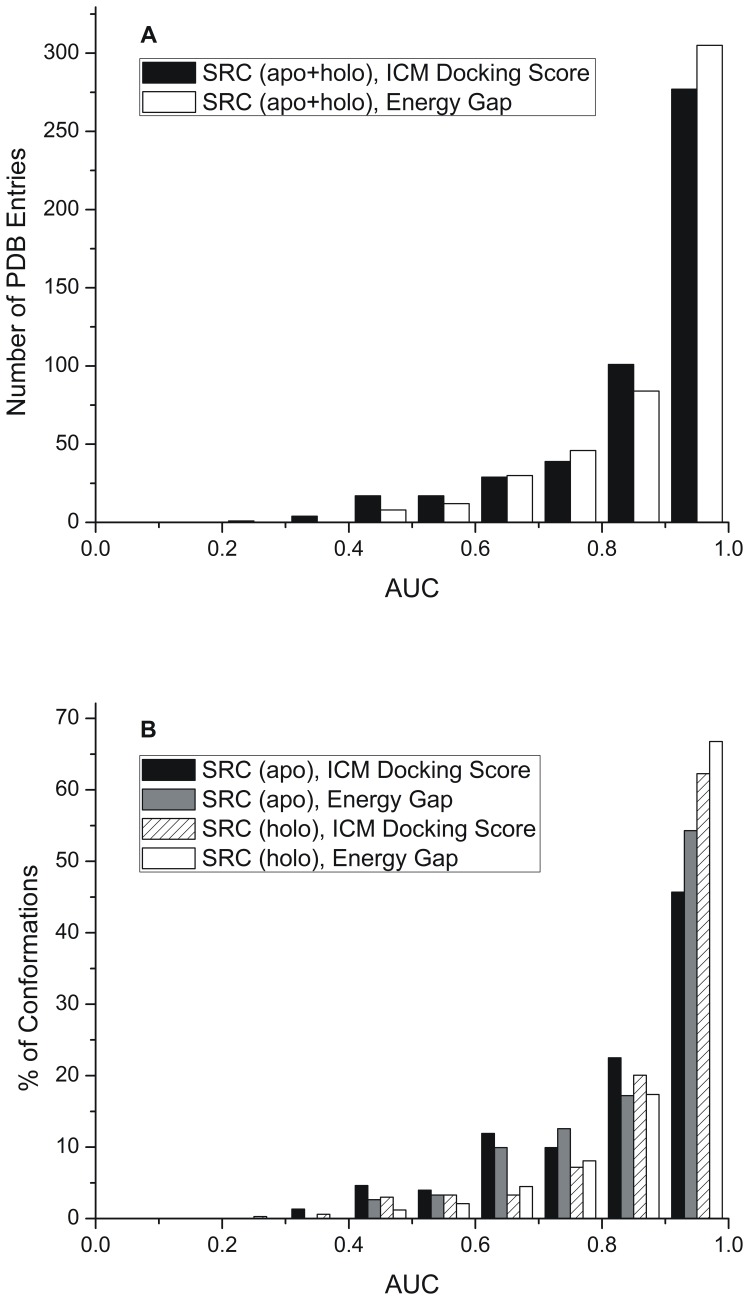
Distributions of AUC values obtained from single-receptor conformation VS. (A) for 485 protein conformations included in the Pocketome benchmark and (B) for 485 protein conformations split in 334 holo and 151 apo structures. See Tables S1, S2, S3 for details.

First, the energy gap was evaluated for its ability to distinguish true binders from decoys for each 485 single receptor conformation, included in the Pocketome benchmark, individually. The performances of the energy gap and the best docking score were measured as the area under the ROC curve, or ROC AUC (see Materials and Methods section). The resulting distributions of AUC values are presented in Figure 2. The analysis of the histograms of AUC values obtained when energy gap is chosen as a discrimination measure shows that 98% of individual structures had favorable AUC values greater than 0.5, 96% had AUC values greater than 0.6, 90% >0.7, 80% >0.8, and 63% of individual structures displayed good recognition with AUC values greater than 0.9 (Figure 2A). The analysis of the histogram of AUC values obtained when the best docking score is chosen as a discrimination measure shows that 95% of individual structures had favorable AUC values greater than 0.5, 92% had AUC values greater than 0.6, 86% >0.7, 78% >0.8, and 57% of individual structures displayed good recognition with AUC values greater than 0.9 (Figure 2A).

Previously, in [47], where the best docking score was chosen to calculate AUC values, the authors reported that “holo conformations have better separation power than apo ones.” When we split the benchmark into holo and apo structures, we observed that, when energy gap is chosen as discrimination measure, the distribution of AUC values for holo structures shifted towards higher AUC values with 67% of holo structures display a good AUC value (>0.9) compared to 54% in case of apo structures (see Figure 2B). The analysis of results obtained when the best docking score is chosen as a discrimination measure shows that 62% holo and 46% apo structures display good recognition with AUC values >0.9. Based on our observations, we can indeed conclude that holo structures perform better than apo ones. The benefit of considering both holo and apo structures is that it increases the diversity of binding pockets, which are required for the recognition of a specific binding profile [47]. According to our results, the energy gap is more successful at separating the binders from decoys in both holo and apo structures.

### Screening Performance of the Energy Gap: Multiple-Receptor Conformations Virtual Screening

**Figure 3:**
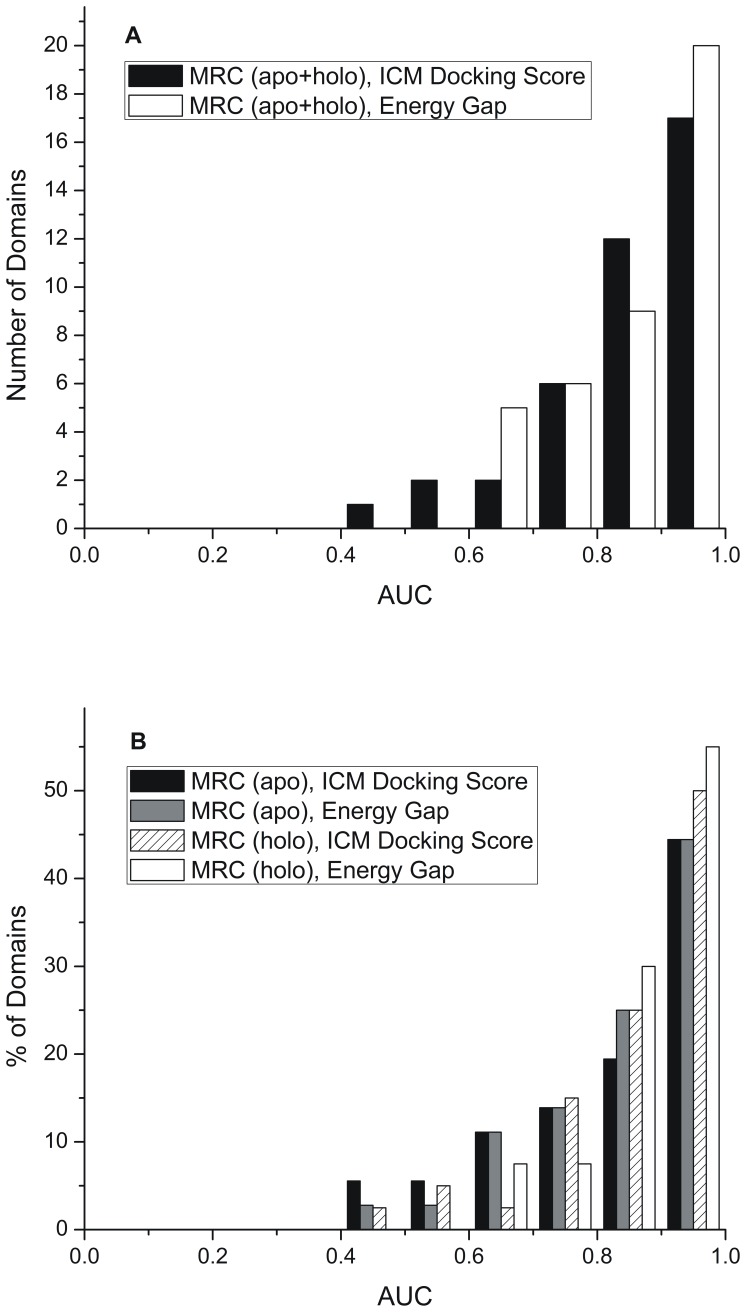
Distributions of AUC values obtained from multiple-receptor conformation VS. (A) for 40 protein domains included in the Pocketome benchmark and (B) for 40 protein domains split in 40 holo and 36 apo domains. See Tables S4, S5, S6 for details.

Consideration of the receptor flexibility is crucial for structure-based drug design and VS methodologies [34], [47]–[59]. Multiple-Receptor Conformations (MRC) are a practical alternative to mimic receptor flexibility [34], [47], [48], [51], [52], [56], [57]. To further study the recognition properties of the energy gap and influence of the receptor flexibility on the performance of the energy gap, we incorporated receptor flexibility by combining 485 protein X-ray conformations, included in the Pocketome benchmark, in 40 protein domains. On average, the 40 protein domains contained about 12 conformations (4 of them apo and 8 holo) and 8 ligands each. For each protein domain, AUC values were evaluated, and performances of the energy gap and the best docking score were compared to each other. A histogram analysis when the energy gap is chosen as a discrimination measure shows that all of the protein domains display AUC values greater than 0.6, 87% display AUC values greater than 0.7, 72% >0.8, and 50% of protein domains displayed good recognition with AUC value greater than 0.9 (Figure 3A). The analysis of the histograms of AUC values obtained when the best docking score is chosen as a discrimination measure shows that 97% of protein domains had favorable AUC values greater than 0.5, 92% had AUC values greater than 0.6, 87% >0.7, 72% >0.8, and only 42% of protein domains displayed good recognition with AUC value greater than 0.9 (Figure 3A). The resulting AUC value distributions for apo and holo ensembles are presented in Figure 3b. The analysis of the histograms, when the energy gap is chosen as discrimination measure, shows that 55% of holo domains display good AUC values (>0.9) compared to 44% in the case of apo domains (see Figure 3B). The analysis of results obtained when the best docking score is chosen as a discrimination measure show that 50% holo and 44% apo ensembles display good recognition with AUC values >0.9 (see Figure 3B).

**Figure 4:**
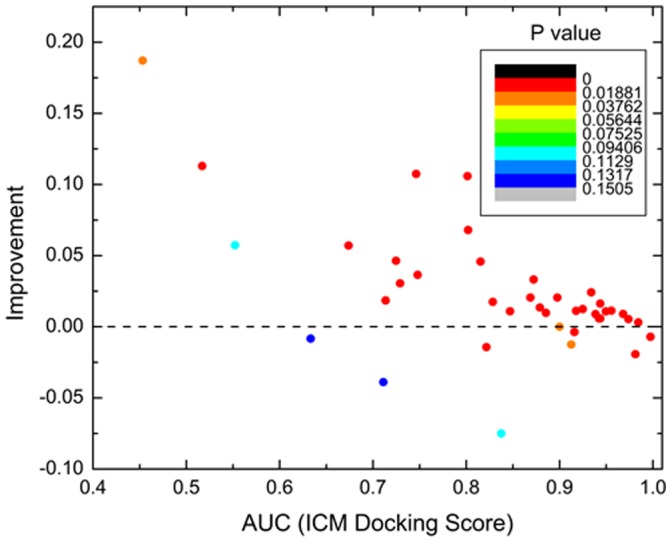
Color-coded dependence of achieved improvement in ROC AUC values obtained by the energy gap on original ROC AUC values obtained by docking score. Each dot represents one protein domain. The dots are colored in different colors spread evenly among P-values from 0.00 to 1.00 (smallest P-values, that is, most statistically significant are red, highest P-values are blue). Overall improvement is achieved in 77% of cases in binder/decoy ligand discrimination. In 14% of cases the energy gap and the docking score performed equally. The improvement is particularly high (up to 0.19 AUC units) for the previously problematic cases with original AUC value below 0.9. In fact for these cases we obtained improvement in 83% of the proteins domains.

In Figure 4, the performance of the energy gap is presented for each of the 40 protein domains. In this figure, AUC values obtained when the energy gap is chosen as a discrimination measure were compared to AUC values obtained when the best docking score is chosen as such a measure.

By using the energy gap, the binder/decoy discrimination ROC AUC value was improved for 77% protein domains when compared to the performance of the best docking score. The improvement was particularly high for the previously problematic cases (weak performance of the best docking score) with original AUC values below 0.9. For these cases we obtained improvement in 83% of protein domains (Figure 4).

Furthermore, the performance of the energy gap in true binders/decoys discrimination was evaluated through MRC VS against 468 high resolution X-ray protein conformations of 36 pharmaceutically relevant protein targets from the Directory of Useful Decoys (DUD), which is one of the most challenging test sets for benchmarking VS. The biggest challenge comes from the fact that true binders included in DUD are selected based on experimental activity and independently from receptor X-ray structures. In addition to that, decoys included in DUD are “*bona-fide*” [34] non-binders, since there is no experimental evidence regarding their lack of activity. For each protein target, AUC values were evaluated, and performances of the energy gap and the best docking score were compared to each other (see Table 1). By using the energy gap, the binder/decoy discrimination ROC AUC value was improved for 64% protein targets when compared to the performance of the best docking score. Notably, the highest improvement was obtained for previously reported problematic protein target HMDH_HUMAN [34]. For this protein target we obtained improvement of 0.14 AUC units. Statistical analysis showed that the significance of these results was P-value <10^−7^.

**Table 1:** The performance of the energy gap obtained from MRC VS for 36 protein targets included in the DUD benchmark.

Protein Target	AUC: Best Docking Score	AUC: Energy Gap	Early Recognition Metric: Best Docking Score	Early Recognition Metric: Energy Gap
P12821_ACE_HUMAN	0.6275	0.6329	0.6327	0.571
P04058_ACES_TORCA	0.7574	0.747	0.7143	0.667
P56658_ADA_BOVIN	0.4339	0.5589	0.4348	0.478
P15121_ALDR_HUMAN	0.5985	0.5912	0.6154	0.654
P00811_AMPC_ECOLI	0.6717	0.6959	0.4762	0.476
P10275_ANDR_HUMAN	0.6405	0.6963	0.7162	0.627
P24941_CDK2_HUMAN	0.7305	0.7209	0.7	0.66
P22734_COMT_RAT	0.6868	0.647	0.6364	0.636
P00374_DYR_HUMAN	0.8925	0.9198	0.9104	0.93
P00533_EGFR_HUMAN	0.8599	0.8775	0.8446	0.849
P03372_ESR1_AG_HUMAN	0.8155	0.7259	0.6866	0.642
P03372_ESR1_ANT_HUMAN	0.7589	0.7447	0.8462	0.744
P00742_FA10_HUMAN	0.9023	0.8872	0.831	0.789
P11362_FGFR1_HUMAN	0.4802	0.477	0.5254	0.542
P04150_GCR_HUMAN	0.4759	0.5836	0.3205	0.462
P04035_HMDH_HUMAN	0.5798	0.7175	0.6571	0.714
P07900_HS90A_HUMAN	0.646	0.7101	0.5417	0.625
P0A5Y6_INHA_MYCTU	0.4647	0.5566	0.4118	0.424
P03176_KITH_HHV11	0.6285	0.621	0.6364	0.591
P08235_MCR_HUMAN	0.8005	0.879	0.6667	0.778
P47811_MK14_MOUSE	0.4877	0.5206	0.5078	0.563
P27907_NRAM_INBBE	0.881	0.8148	0.9388	0.816
P26446_PARP1_CHICK	0.7871	0.8425	0.7273	0.758
O76074_PDE5A_HUMAN	0.6914	0.704	0.6078	0.608
P05979_PGH1_SHEEP	0.7322	0.7352	0.68	0.63
Q05769_PGH2_MOUSE	0.5855	0.5585	0.5632	0.566
P55859_PNPH_BOVIN	0.6689	0.6469	0.76	0.76
P03366_POL_HV1B1	0.6307	0.667	0.55	0.575
P06401_PRGR_HUMAN	0.7008	0.7245	0.6667	0.667
P08179_PUR3_ECOLI	0.9209	0.9241	0.8571	0.857
P00489_PYGM_RABIT	0.563	0.6034	0.7308	0.731
P19793_RXRA_HUMAN	0.7829	0.7849	0.8	0.85
P12931_SRC_HUMAN	0.6973	0.7219	0.6774	0.684
P00734_THRB_HUMAN	0.793	0.7947	0.8308	0.8
P00760_TRY1_BOVIN	0.9009	0.8867	0.8636	0.886
P35968_VGFR2_HUMAN	0.7031	0.7189	0.6757	0.703

For comparison purposes, the performance of the best docking score is presented.

Because successful VS ranks true binders early [60], [61], the early recognition metric based on the energy gap and the best docking score was evaluated for each protein target as it is described in the Materials and Methods section. In Table 1, the fractions of true binders recovered within *N* top-ranked hits sorted by the energy gap and the best docking score, where *N* is the number of true binders for a given protein target, are presented. Obtained results indicate that the energy gap rewards early recognition better compared to the best docking score in 50% of protein targets and in another 19% of protein targets the energy gap and the best docking score reward early recognition identically. These results certainly indicate that the energy gap outperforms the best docking score in its discrimination performance.

### The Height of the Native Binding Phase

**Figure 5:**
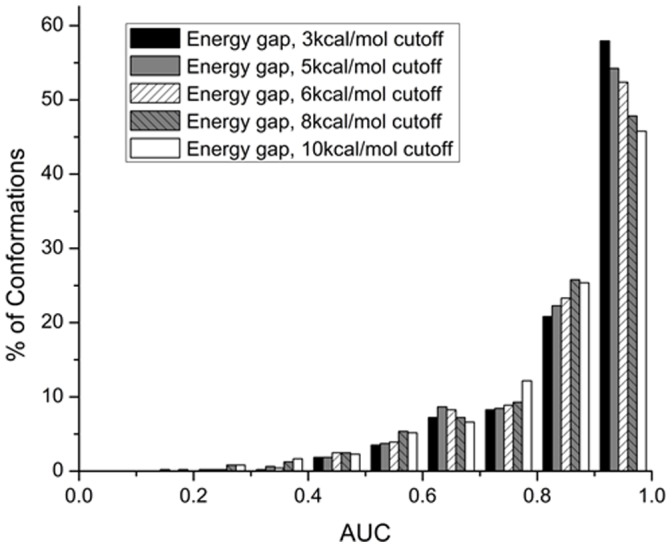
Distribution of AUC values obtained from single-receptor conformation VS for 485 protein conformations included in the Pocketome benchmark when different energy cutoffs from the best docking score were applied to cluster binding conformations within the native binding phase. See Table S7 for details.

The folding landscape of proteins has two major phases, a) molten globule phase where the system has high entropy and energy, and b) native phase where the system has low entropy and energy [7]. Similarly, the bimolecular binding energy landscape also has two major phases, a) non-native unbinding phase, and b) native binding phase. Native binding phase is a cluster of near native binding conformations and the native binding state [20], [21]. Here we used the energy gap as a descriptor to systematically analyze and measure the height of the native binding phase for all 485 X-ray protein conformations included in the Pocketome benchmark. In the present study, we defined the energy gap as the difference between the lowest energy pose and the average energy of all other generated poses. In order to measure the height of the native binding phase we applied different energy cutoffs (from the best docking score) to cluster the binding conformations within the native binding phase. We then calculated the energy gap as the difference between the average energy of all binding conformations within this cluster (included the best docking score) and the average energy of all other conformations, and evaluated the performance of this energy gap for its ability to distinguish true binders from decoys as it is described above. The results of the energy gap performance when different energy cutoffs were applied are shown in Figure 5 and Table 2.

**Table 2:** The performance of the energy gap obtained from single-receptor conformation VS for 485 X-ray protein conformations included in the Pocketome benchmark when different energy cutoffs from the best docking score were applied to cluster binding conformations within the native binding phase.

Energy cutoff (kcal/mol)^a^	AUC >0.5	AUC >0.6	AUC>0.7	AUC >0.8	AUC >0.9
3.0	98%	94%	87%	79%	60%
4.0	98%	94%	86%	77%	56%
5.0	97%	94%	85%	76%	54%
6.0	97%	93%	85%	75%	52%
***7.0***	***96%***	***91%***	***84%***	***75%***	***50%***
8.0	95%	90%	83%	74%	48%
9.0	95%	90%	83%	72%	47%

Percentage of protein conformations displaying AUC values above certain energy cutoffs is presented. See Table S7 for details.

a According to ICM scoring function.

We consider the case when half or more of the X-ray structures included in the dataset show AUC values greater than 0.9 as an indicator of a good performance. As it can be seen in Figure 5 and Table 2, the energy gap, when energy cutoff was set up to 7.0 kcal/mol, still shows a good performance. This finding leads to the conclusion that on average the native binding phase extends to 7.0 kcal/mol height from the best docking score. Interestingly, this result is in good agreement with study done by Shan et al. [30], where unguided molecular dynamics simulations of the entire binding process of the cancer drug dasatinib and the kinase inhibitor PP1 to Src protein kinase were conducted and an energy landscape analysis was performed (see Figure 1C in [30]). Furthermore, we calculated an average value of maximum heavy atom RMSDs from the best docking pose to all conformations within the native binding phase for all true binders of the entire benchmark. We found that on average the width of the native binding phase is approximately equal to 6.0 Å RMSD from the lowest energy pose. Obtained results are consistent with previously published studies, where Verkhivker et al. [20] found that the width of the native binding phase for the methotrexate-dihydrofolate reductase system extends to nearly 5.0 Å RMSD from the native structure, and Wei et al. [29] found that the width of the native binding phase extends to 6.5 Å and 8 Å RMSDs for neuraminidase and cyclooxygenase-2 respectively.

### Consideration of the Binding Conformations within Boundaries of the Native Binding Phase Significantly Increases Success Rate of Near Native Binding Pose Identification

**Figure 6:**
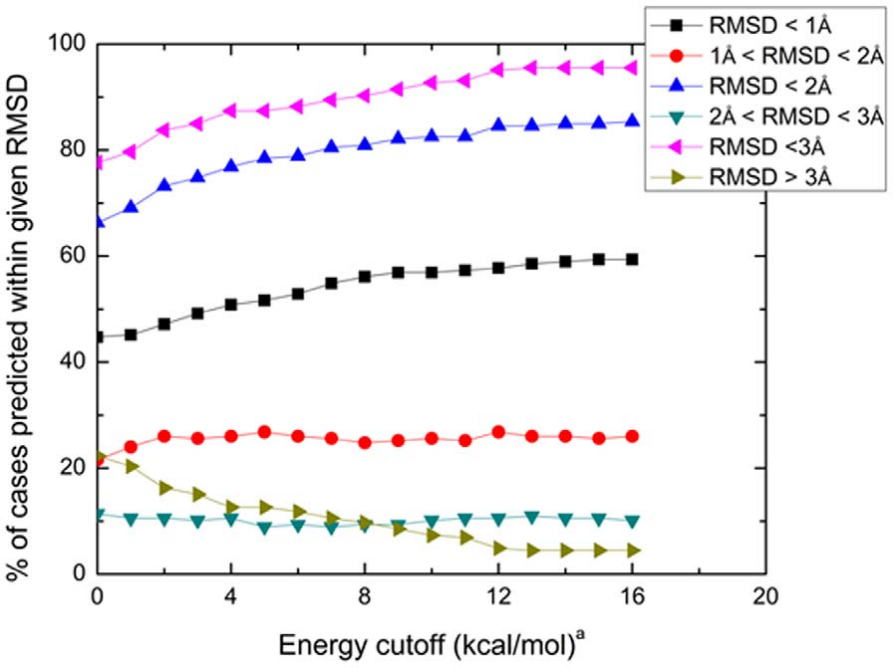
Changes in the success rate of finding the correct X-ray binding mode as a function of energy cutoff. ^a^According to ICM scoring function.

The prediction of the ligand native binding pose is an essential problem in drug discovery. As we mentioned in the introduction, literature has already reported that the correct binding pose is often not the best scored solution, and consideration of a small set of representative docking solutions significantly increases the success rate of near native binding pose identification [26]–[28]. However, to the best of our knowledge, the theoretical justification of number of poses to be considered has not yet been proposed. Here we examined and statistically quantified the success rate of near native binding pose identification (heavy atom RMSD to the crystallographic pose is under 2.0 Å) when the binding conformations within different energy cutoffs from the best docking score have been considered. For a given true binder, we applied different energy cutoffs from the best docking pose to draw the boundaries of the binding conformations to be considered. Furthermore, for all conformations within these boundaries, we calculated the heavy atom RMSDs from the crystallographic pose, and the closest conformation with its corresponding heavy atom RMSD was saved. These RMSD values were compared to the RMSD values between the lowest energy poses (Energy cutoff  = 0) and the crystallographic poses (Figure 6). As it can be seen in Figure 6, the success rate of near native binding pose identification increases substantially when all binding conformations within the boundaries of the native binding phase (energy cutoff  = 7.0 kcal/mol) are considered compared when only the lowest energy poses were considered. In fact, we found that 80% of the time, correct, near native poses were identified when all docking conformations within the boundaries of the native binding phase were considered as opposed to 66% when only the lowest energy poses were considered. We observed that the increase in success rate of near native binding pose identification is mostly due to the decrease in the number of cases with an RMSD greater than 3.0 Å. Moreover, our findings indicate that 78% of the time, the first near native docking solutions obtained among all generated binding conformations within the boundaries of the native binding phase are ranked within the first five scored poses and in 80% of the time within the first ten scored poses. Expanding the boundaries of the binding conformations to be considered beyond the energy cutoff of 7.0 kcal/mol improves the success rate of near native binding pose identification (see Figure 6). We found that on average the energy difference between the lowest energy pose and the first near native pose is approximately equal to 7.8 kcal/mol. However, the added complexity of having more than five docking poses to consider may not be justified for routine VS experiments. Obtained results clearly suggest that consideration of the binding conformations within the boundaries of the native binding phase increases the success rate of near native geometry identification.

### Evaluation of the Energy Gap Performance on PERK Inhibitors Designed using Homology Modeling

**Figure 7:**
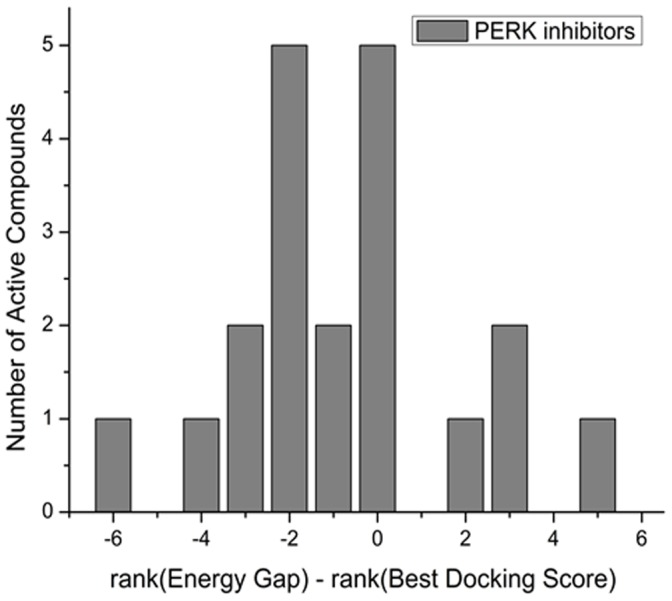
Histogram of the difference in the ranking of PERK inhibitors by the energy gap and the best docking score. “0” indicates no change in ranking by the energy gap as compared to the best docking score: for example, “0” means that ligand X was the 11^th^ ranked compound in the list by the energy gap and also the 11^th^ ranked compound in the list by the best docking score. Negative numbers mean that the energy gap ranking is higher than the best docking score ranking: e.g. if the compound is ranked 5^th^ in the list by the energy gap and 7^th^ in the list by the best docking score the above score would be −2. The histogram shows many more compounds with negative difference scores showing that the energy gap results in a higher true positive yield upon experimental testing of the top N compounds in this case of VS against protein homology models that was independent of the set in this study.

To further evaluate the discrimination ability of the energy gap, we tested its performance on a more sophisticated case: VS against a protein homology model. We applied the binding energy landscape analysis to PERK inhibitors that were recently discovered using homology models [35]. For each of 32 compounds, which included 20 active and 12 inactive compounds, we calculated the energy gap and the best binding score. We then ranked the compounds according to the energy gap and the best docking score, respectively. As expected, the energy gap is superior to the best docking score in its ability to more highly rank active compounds from inactive ones (Figure 7). In fact, in 11 out of 20 cases (55%) the energy gap ranks active compounds higher than the best docking score; in another 5 cases (25%) both approaches performed identically, and in only 4 cases (20%) the best docking score ranks active compounds higher than the energy gap. These results indicate that the binding energy landscape analysis is able to improve the hits-list processing in the VS against protein homology models.

### Future Directions

All of the docking experiments in this paper were performed with ICM, and logical questions could arise about the applicability of obtained results to other platforms. Consistency between our findings and results obtained in independent studies [29] and [30] clearly suggest that all of our principle observations are transferable to other platforms and that incorporating information of the binding energy landscape greatly benefits the process of VS. However, it is certain that more systematic studies across different docking programs and scoring functions are necessary. Also, it is important to mention that in this study protein flexibility was modeled by using multiple X-ray conformations. Although this approach is one of the most promising alternatives to mimic protein plasticity, it has limitations. Among those limitations we would like to mention the lack of multiple experimental structures for many of protein targets. In addition to that, pre-generated receptor conformational ensemble may not catch receptor conformational changes upon ligands binding to receptor [58]. Usage of flexible docking approaches, such as MedusaDock [58] and RosettaLigand [59], may potentially help to overcome the limitations mentioned above. We hope that our study will stimulate research in these areas.

There is no doubt that it is difficult to avoid errors when evaluating the binding energy levels corresponding to different docking poses [5]. This automatically raises the questions of which docking poses/energy levels exist in reality and which do not. The success of applying the energy landscape theory to the rational drug discovery depends on our ability to filter (geometrically and energetically) the subset of representative realistic docking poses from the entire set of generated solutions.

### Conclusions

In this study we investigated the screening performance of the energy gap between the lowest energy (best scored pose) and the average energy of all other poses scored in the searched binding energy landscape to discriminate true binders from decoys. We performed the study based on the assumption that the energy gap calculated during docking is a measure of the binding energy landscape sharpness, an assumption which was previously proposed and supported [21], [22]. To test the performance of the energy gap, we performed single and ensemble docking experiments in two large and diverse benchmarks of therapeutically relevant proteins. We found that, despite the fact that current ICM docking score already displays good discrimination power between true binders and decoys, the energy gap outperforms (improvement in AUC values in 77% of protein domains for multiple-receptor conformations VS experiments included in the Pocketome benchmark and in 64% of protein targets included DUD benchmark) the ICM docking score in its ability to discriminate true biologically relevant binders among decoys. Moreover, true binders tend to have larger energy gaps than decoys. We also found that the energy gap rewards early recognition better compared to the best docking score. Furthermore, we used the energy gap as a descriptor to evaluate the height of the native binding phase and found that it has about 7.0 kcal/mol height from the lowest energy pose. We tested the success rate of near native binding pose identification when the binding conformations within the boundaries of the native binding phase have been considered. We observed that the success rate increases from 66% when only the lowest energy conformations were considered to 80% when all conformations within the native binding phase were considered. We also found that expanding the boundaries of the binding conformations to be considered beyond the energy cutoff of 7.0 kcal/mol (native binding phase) increases the success rate of near native binding pose identification, however the added complexity of having more than five docking poses to consider may not be justified for routine VS experiments. Finally, we applied the binding energy landscape analysis to the PERK inhibitors recently discovered in our laboratory using homology modeling. The results showed that the energy gap is superior to the best docking score in its ability to discriminate the active compounds from the inactive ones on the VS against not only crystal structures but also protein homology models.

VS is based on the assumption that the final state of the ligand-receptor complex contains all the necessary information for the discrimination of biologically relevant ligand-receptor complex conformations from biologically artifactual alternatives. Notably, a ligand may show excellent complementarity with a receptor target pocket *in silico* but may be biologically irrelevant due to its statistical mechanics profile: it may have a preference for many sites on the receptor and on other receptors at once. The presence of a funnel-shaped energy landscape, detectable by an energy gap as we have defined it here, may discriminate these artifactual ligands from physiologically relevant ligands exhibiting the same level of *in silico* complementarity in their final bound state. Based on obtained results, we can conclude that the energy gap reported by VS algorithms contains information distinguishing between these two scenarios. The successful integration of binding energy landscape scores (such as the energy gap investigated in this study) with the usual VS lowest energy score can improve the physiological relevance of well established approaches in VS for rational lead discovery, which are often adequate in discriminating binders from the background but generate many false positives. From this study, we suggest that in VS experiments the binding energy landscape analysis should be applied after all clearly structurally incompatible chemicals are filtered based on the lowest energy score. After this step all binding conformations within the boundaries of the native binding phase have to be investigated and analyzed in detail.

## Supporting Information

Table S1 **PDB IDs, Swiss-Prot IDs and performance of 485 X-ray protein conformations included in the Pocketome benchmark.**
(XLS)

Table S2 **PDB IDs, Swiss-Prot IDs and performance of 334 holo X-ray protein conformations included in the Pocketome benchmark.**
(XLS)

Table S3 **PDB IDs, Swiss-Prot IDs and performance of 151 apo X-ray protein conformations included in the Pocketome benchmark.**
(XLS)

Table S4 **Swiss-Prot IDs and performance of 40 protein domains included in the Pocketome benchmark.**
(XLS)

Table S5 **Swiss-Prot IDs and performance of 40 protein domains when only holo structures included in the Pocketome benchmark were considered.**
(XLS)

Table S6 **Swiss-Prot IDs and performance of 36 protein domains when only apo structures included in the Pocketome benchmark were considered.**
(XLS)

Table S7 **The performance of the energy gap obtained from single-receptor conformation VS for 485 X-ray protein conformations included in the Pocketome benchmark when different energy cutoffs from the best docking score were applied to cluster binding conformations within the native binding phase.**
(XLS)

Table S8 **Complete MRC VS DUD benchmark.**
(XLS)
